# The bacterial pathogen and resistance spectrum in a dermatological inpatient ward: a six-year, retrospective, epidemiological study

**DOI:** 10.3205/dgkh000419

**Published:** 2022-09-02

**Authors:** Philipp Stelzhammer, Wolfgang Weber, Hermine Binder, Ulrich Sagel, Christoph Aspöck, Franz Trautinger

**Affiliations:** 1 Department of Dermatology and Venereology, University Hospital St. Poelten, St. Poelten, Austria; 2 Karl Landsteiner Institute of Dermatological Research, St. Poelten, Austria; 3 Institute for Hygiene and Microbiology, University Hospital St. Poelten, Karl Landsteiner Private University of Health Sciences, St. Poelten, Austria; 4 Hospital Pharmacy, University Hospital St. Poelten, St. Poelten, Austria; 5 LADR MVZ Dres. Bachg, Haselhorst & colleagues, Recklinghausen, Germany

**Keywords:** dermatology, skin and soft tissue infections, antibiotic consumption

## Abstract

**Aim:**

Treatment of bacterial soft tissue infections is an essential part of clinical dermatology, and the choice of antibiotic therapy is often empirical. The aim of this longitudinal retrospective study was to evaluate bacterial epidemiology, resistance patterns and antibiotic consumption in a dermatological inpatient ward.

**Method:**

Bacterial isolates and antimicrobial susceptibility testing from a dermatological inpatient ward were recorded retrospectively from 2011 to 2016. The antibiotic consumption was evaluated and given as the assumed defined daily dose [DDD] per 100 days of covering per year.

**Results:**

A total of 4,800 bacterial isolates were included (skin, mucous membrane and wounds 87%, urine 9.5%, blood 1.7%, tissue and tissue fluids 1.6%). The proportion of Gram-positive bacteria was 58% (*Staphylococcus aureus* 37.8%, *coagulase-negative staphylococci* 21.5%, *Enterococcus spp*. 16.7%). *Pseudomonas aeruginosa* (27.2%), *Escherichia coli* (17.5%) and *Proteus spp*. (13.1%) were the most common Gram-negative bacteria. The proportion of multi-resistant pathogens was 5.8% for *methicillin-resistant S. aureus*, 0.9%, 0.8% and 1.8% for* multi-resistant P. aeruginosa*, *ESBL-producing E. coli* and E*SBL-producing Klebsiella pneumoniae* of all isolates. Beta-lactam antibiotics were the most used drugs (14.4, 10.8, and 9.6 DDD/100 for aminopenicillins, cefalexin, and penicillin G), followed by clindamycin (9.0 DDD/100 patient days).

**Conclusion:**

In view of the frequency of bacterial soft tissue infections and their need for inpatient treatment with mostly empirically chosen antibiotics, systematic microbiological surveillance should be recommended for dermatological inpatient wards.

## Zusammenfassung

**Zielsetzung:**

Die Behandlung bakterieller Weichteilinfektionen ist ein wesentlicher Bestandteil der klinischen Dermatologie und die Wahl der antibiotischen Therapie erfolgt oft empirisch. Ziel dieser longitudinalen retrospektiven Studie war die Evaluierung der bakteriellen Epidemiologie, des Resistenzverhaltens und des Antibiotikaverbrauchs auf einer dermatologischen Bettenstation.

**Methode:**

Bakterienisolate und Resistogramme einer dermatologischen Bettenstation wurden im Zeitraum von 2011 bis 2016 retrospektiv erfasst. Der Antibiotikaverbrauch wurde ermittelt und als angenommene mittlere Tagesdosis [DDD] pro 100 Belagstage pro Jahr angegeben.

**Ergebnisse:**

Insgesamt wurden 4.800 Bakterienisolate gewonnen (Haut, Schleimhaut und Wunden 87%, Urin 9,5%, Blut 1,7%, Gewebe und Gewebsflüssigkeiten 1,6%). Der Anteil Gram-positiver Bakterien betrug 58% (*Staphylococcus aureus* 37,8%, *Koagulase-negative Staphylokokken* 21,5%, *Enterococcus spp*. 16,7%). *Pseudomonas aeruginosa* (27,2%), *Escherichia coli* (17,5%) und *Proteus spp*. (13,1%) waren die häufigsten Gram-negativen Bakterien. Der Anteil multiresistenter Erreger lag für *Methicillin-resistenten S. aureus* bei 5,8%, für *multiresistenten* *P. aeruginosa*, *ESBL-bildende* *E. coli* und *ESBL-bildende* *Klebsiella pneumoniae* bei 0,9%, 0,8% und 1,8% aller Isolate. Betalaktamantibiotika waren die häufigsten verwendeten Medikamente (14,4, 10,8 und 9,6 DDD/100 bei Aminopenicillinen, Cefalexin und Penicillin G), gefolgt von Clindamycin 9,0 DDD/100 Belegungstage.

**Schlussfolgerung:**

In Anbetracht der Häufigkeit bakterieller Weichteilinfektionen und ihres Bedarfs für stationäre Behandlung bei meist empirischer Antibiotikaauswahl sollte auch für dermatologische Bettenstationen eine systematische mikrobiologische Überwachung empfohlen werden.

## Introduction

Skin and soft tissue infections [SSTI] are among the most common reasons for outpatient clinic visits and for inpatient hospital treatment [1], [2], [3], [4]. 

The majority of SSTIs are caused by Gram-positive cocci, especially *Staphylococcus* *aureus* and *Streptococcus pyogenes*. Among the Gram-negative germs, *Pseudomonas aeruginosa* and *Escherichia coli* are particularly relevant [5], [6], [7]. According to a recommendation by the Infectious Diseases Society of America, SSTIs are divided into non-purulent (erysipelas, necrotizing soft tissue infections) and purulent (furuncles, carbuncles, abscesses), which in turn are classified as mild, moderate or severe depending on their characteristics and course [8]. In a previous classification, a distinction was made between uncomplicated and complicated SSTIs. Accordingly, all severe infections, surgical site infections, decubitus ulcer, infections of transplants or rather implants and all SSTIs in the presence of diabetes mellitus, chronic liver or kidney dysfunction, peripheral arterial disease, neuropathy, obesity, alcohol or drug abuse and immunosuppression are defined as complicated SSTIs [2], [4]. The therapy of a SSTI consists primarily in pharmacological treatment with systemic antibiotics and surgical intervention.

The increasing prevalence of resistance to the most commonly used antibiotics has become a global problem [9]. Resistance to almost all currently available antibiotic drugs has been observed over the past 70 years. According to the US Centers for Disease Control and Prevention, at least 2.8 million infections with resistant pathogens occurred in 2019, with a mortality of more than 35,000 patients [10]. 

The emergence and spread of resistant bacterial strains is primarily associated with the uncritical consumption of antibiotics and the increasing international mobility of people, animals and goods [10]. According to reports, up to 50% of all prescriptions are made unnecessarily or with an unsuitable antimicrobial agent [11], [12], [13]. Especially when treating infections with nosocomial pathogens including *staphylococci*, *enterococci*, *Pseudomonas spp*. and other Gram-negative rods show increasing resistance problems [14]. Antibiotic resistance not only leads to increased morbidity and mortality, but also has economic and psychosocial consequences, since to an increasing degree newer, more expensive substances have to be used, isolation measures are necessary and, last but not least, fear and stigmatization arise [7]. Knowledge of the specific, local bacterial epidemiology and associated antibiotic resistance can help optimize therapeutic strategies, improve patient outcomes and reduce hospital stays for patients with soft tissue infections [5], [15]. 

Although SSTIs are one of the most common indications for inpatient treatment in dermatological departments, there are no systematic studies of the bacterial spectrum in dermatological inpatient wards. The aim of this longitudinal retrospective study was therefore to record bacterial isolates, the resistance pattern of selected germs and the antibiotic consumption in the two wards at the Department for Dermatology and Venereology at the University Hospital St. Poelten, Austria, between 2011 and 2016.

## Material and method

All smears (including skin, mucous membrane and wound swabs, tissue samples, blood cultures, urine samples and catheter tips) collected between January 1^st^ 2011 and December 31^st^ 2016 as part of routine inpatient diagnostics and bacteriologically analysed at the Department of Hygiene and Microbiology of the University Hospital in accordance with the EUCAST guidelines, were included in the retrospective analysis [16]. According to our internal standards no specific swab technique (such as Levine, Essener Kreisel, etc. [17]) is pre-specified. Due to the retrospective character of the study quality control of wound swabbing was impossible and all samples were included. If several samples were taken from one patient during a continuous stay, only the first positive isolate was included for each type of sample. If there were submissions from a patient from different inpatient stays, each individual inpatient stay was included. 

The antibiotic consumption was determined with data provided by the institutional pharmacy of the University Hospital and presented as the assumed defined daily dose per 100 bed days per year (DDD/100 days bed-days) [18]. Occupancy rates for the inpatient wards were taken from the routine in-house record keeping. 

Descriptive statistics were calculated using Microsoft^®^ Excel^®^ Microsoft 365 MSO (Version 2202) and OriginPro^®^ (OriginLab Corporation, Northampton, MA). 

In the context of this retrospective project, no individual, identifiable, patient-specific data was recorded, and no study-related interventions were carried out. Therefore, no patient-related risks or burdens were associated with the project.

## Results

### Occupancy rates

With a number of 43 beds, inpatient admissions in the study period were 13,063 with a slightly decreasing average length of stay from 6.0 days (2011) to 5.1 days (2016). 12,586 principal diagnoses were recorded and divided into five categories: Skin cancer (50.1%), autoimmune and inflammatory diseases (19.7%), bacterial infections (12.4%), vascular diseases (8.0%), non-bacterial infections (7.3%), and others (2.5%). A detailed list is provided in Table 1 (Tab. 1). 

### Total microbiological spectrum

**Figure 1:**
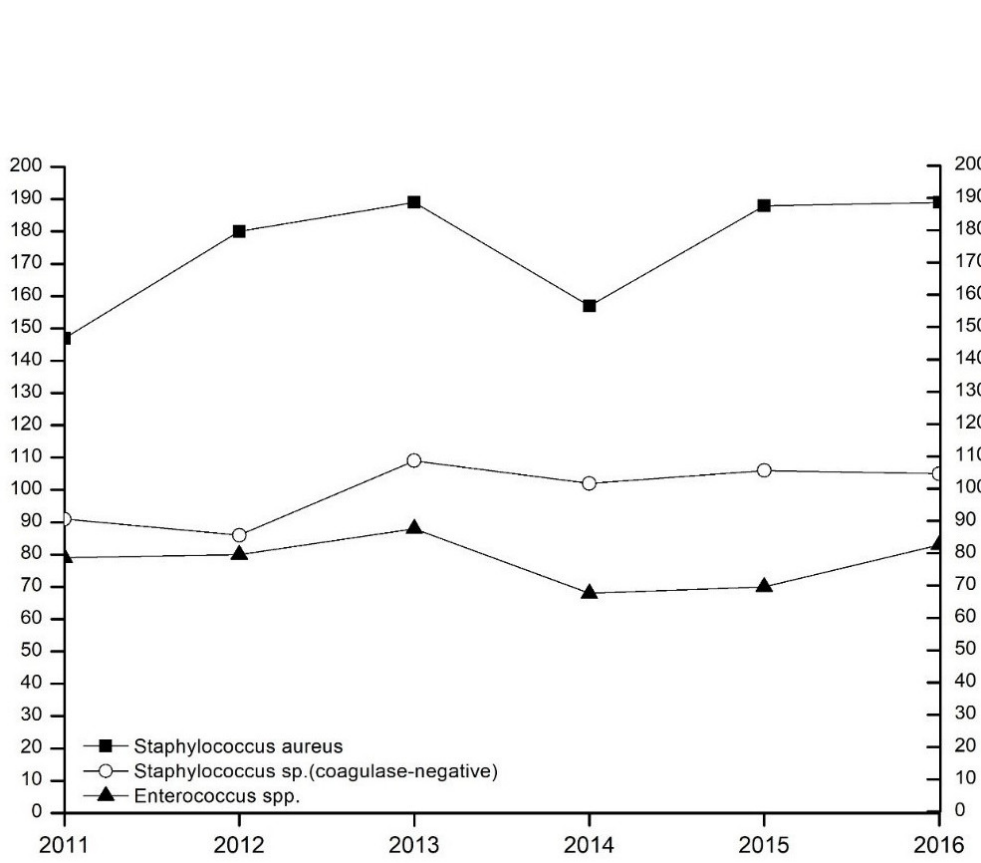
The three most common gram-positive bacteria over time. The ordinate shows the number of respective bacterial isolates (*S. aureus* including *MRSA*). The abscissa indicates the survey year.

**Figure 2:**
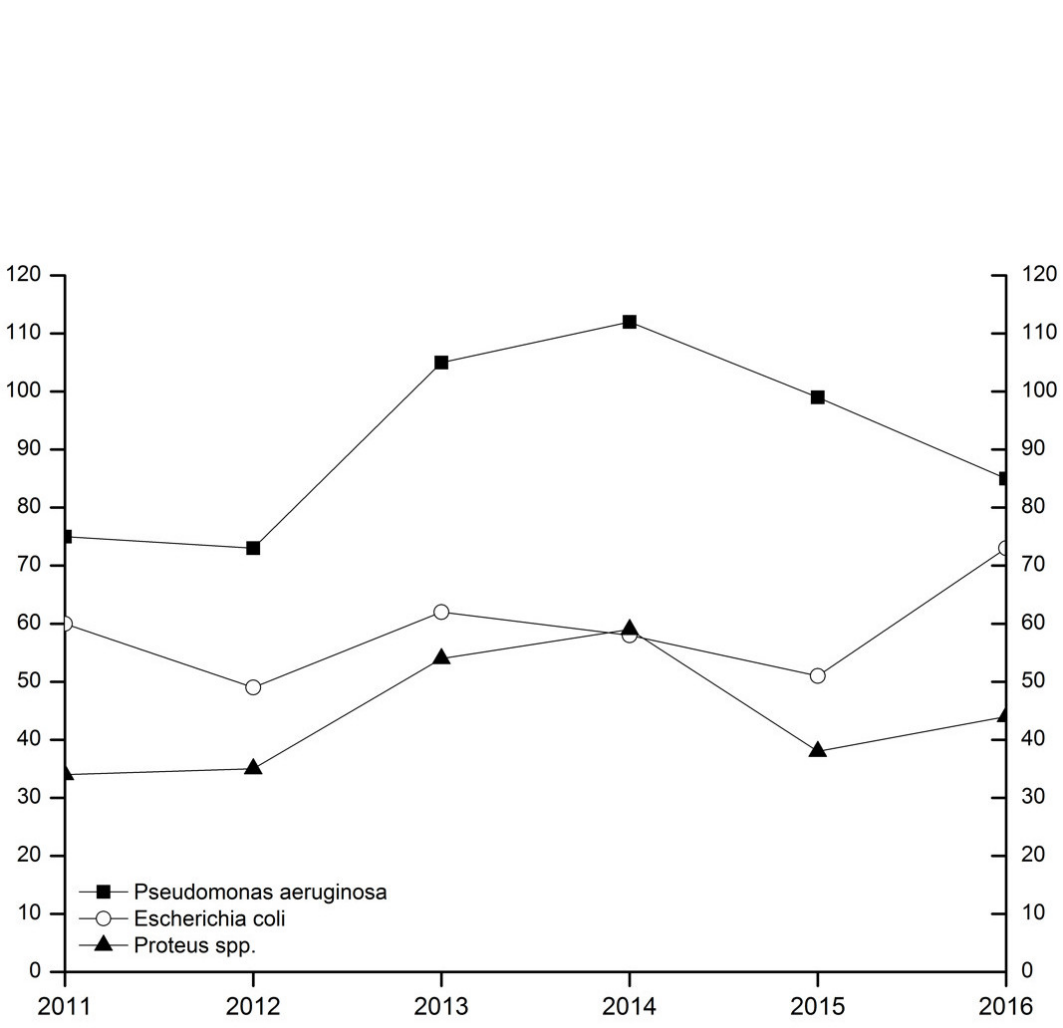
The three most common gram-negative pathogens (time course). The ordinate shows the number of bacterial isolates (*P. aeruginosa* including 3- and 4-MRGN isolates). The abscissa indicates the survey year.

A total of 4,800 bacterial strains were isolated during the study period. 87% of these were obtained from swabs from skin and mucous membranes or wounds, 9.5% from urine samples, 1.7% from blood samples and 1.6% from tissue fluid aspirates. Samples of the oropharynx with only resident flora, urine with anaerobic mixed flora and the accidental fungus were not included in the analyzes. Among the isolates from skin swabs, *S. aureus*, *coagulase-negative staphylococci* and *P. aeruginosa* were the most common representatives. Most of the urine isolates were *E. coli* and *Enterococcus spp*. and in blood cultures yielded *S. aureus* and *S. epidermidis* (Table 2 (Tab. 2)). The percentage of Gram-positive bacteria of all bacterial isolates was 58% with (in descending order of prevalence) *S. aureus*, coagulase-negative *staphylococci* and *Enterococcus spp*. as the most common pathogens. The distribution of the Gram-positive germs remained constant over time (Figure 1 (Fig. 1), Table 3 (Tab. 3)). The proportion of Gram-negative bacteria in all bacterial isolates was 42%. *P. aeruginosa* was found most frequently, followed by *E. coli* and *Proteus spp*. The distribution remained stable over the observation period (Figure 2 (Fig. 2), Table 4 (Tab. 4)).

### Antibiotic resistance 

**Figure 3:**
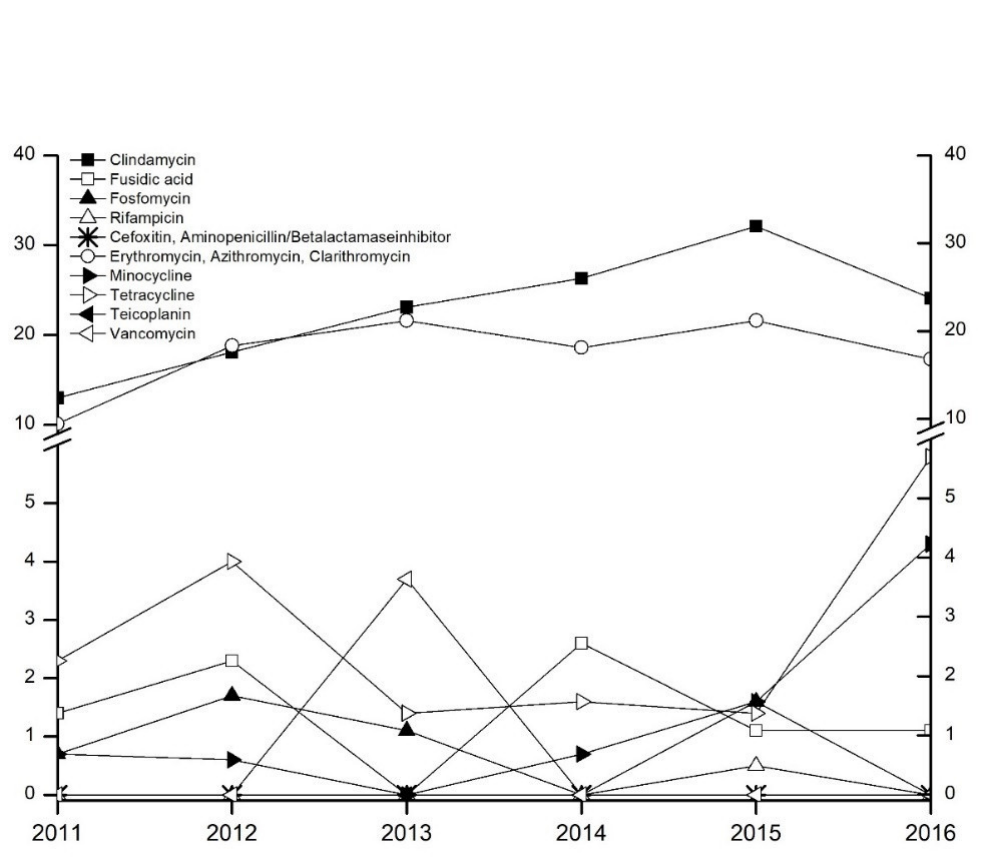
Resistance rates of *S. aureus* including *MRSA* from 2011 to 2016. The axis shows the relative proportion of resistant isolates in relation to the total number of isolates tested in percentage points. The abscissa indicates the survey year.

Resistance to macrolides and clindamycin was most frequently found in *S. aureus*, with the latter tending to increase (Figure 3 (Fig. 3)). The incidence of *methicillin-resistant* *S. aureus* (*MRSA*) was consistently low [absolute number of isolates and total MRSA rate (%): 2011: 9 (6.1%), 2012: 31 (7.3%), 2013: 23 (4.7%), 2014: 26 (5.9%), 2015: 40 (8.2%), 2016: 32 (6%)]. None of the *MRSA* isolates were resistant to linezolid, vancomycin, teicoplanin, or rifampicin and the resistance rates for fosfomycin and fusidic acid were very low. 

**Figure 4:**
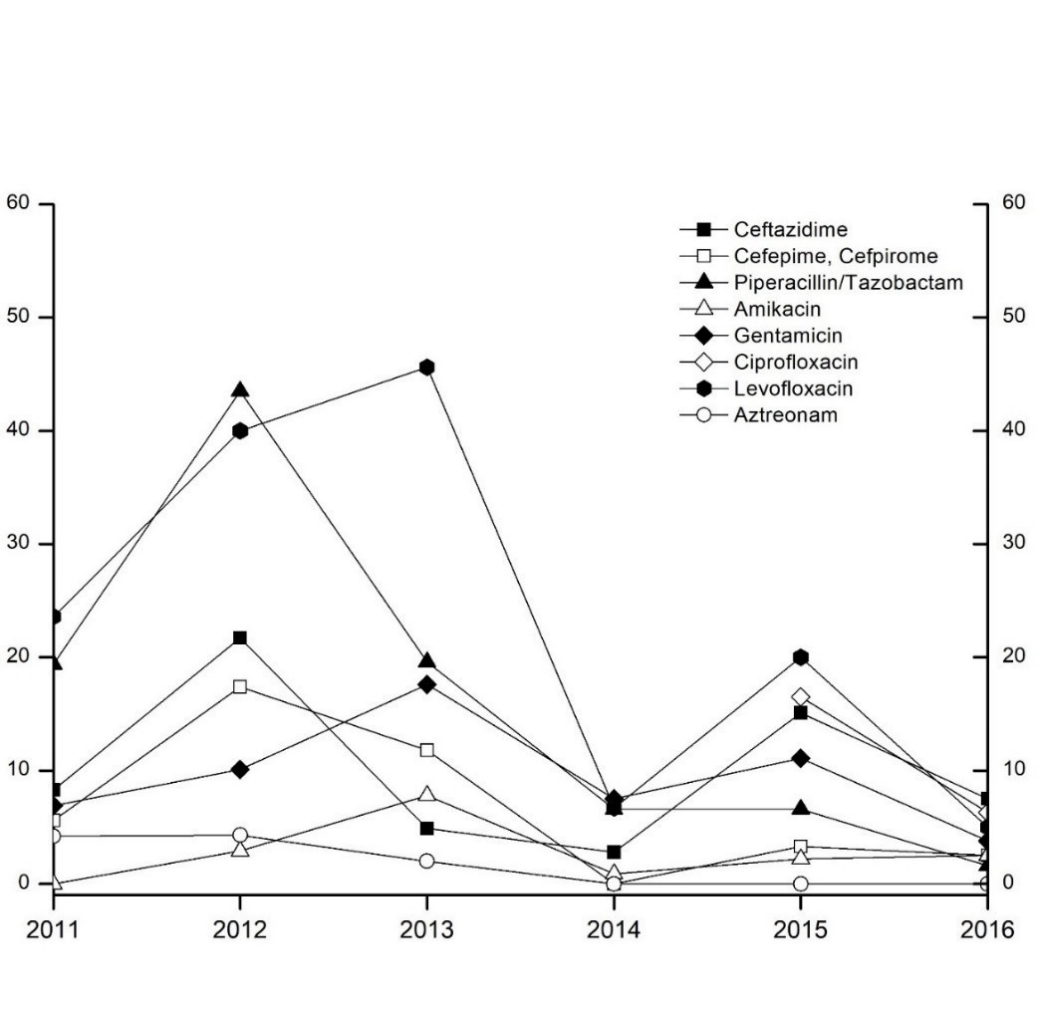
Resistance rates of *P. aeruginosa* from 2011–2016. The ordinate shows the relative resistance share of all tested isolates in percent. The cumulative rates for the 4-MRGN and 3-MRGN isolates (17 isolates) were 100% for piperacillin/tazobactam, 70.59% for imipenem, 86.67% for meropenem, 47.06% for ceftazidime, and 63.64% for ciprofloxacin.

*P. aeruginosa* including 3- and 4-multiresistant Gram-negative isolates (3- and 4-MRGN) frequently showed resistance to levofloxacin and piperacillin/tazobactam at the beginning of the study period (Figure 4 (Fig. 4)). Over the course of time, there were strong fluctuations in the resistance behavior of *P. aeruginosa*. The 3- and 4-MRGN isolates [absolute number of isolates and total 3- and 4-MRGN rate (%): 2011: 0, 2012: 0 (0%), 2013: 1 (0.3%), 2014: 4 (1.1%), 2015: 8 (2.5%), 2016: 4 (1%)] were mainly resistant to piperacillin/tazobactam, but also to carbapenems and quinolones. 

A substantial rate of resistance of *E. coli* including ESBL-forming isolates against aminopenicillins was detected, with sensitivity to trimethoprim and aminopenicillin plus beta-lactamase inhibitor (BLI) maintained (Table 5 (Tab. 5)). Within the ESBL-forming isolates, the resistance rate for cefepime/cefpirome was about 50%, and low for nitrofurantoin and fosfomycin. There were no isolates resistant to carbapenem and mecillinam. The absolute number and total rate of *ESBL-producing* *E. coli* isolates was as follows: 2011: 3 (1%), 2012: 8 (3%), 2013: 9 (2.4%), 2014: 3 (0.8%), 2015: 4 (1.2%), 2016: 10 (2.5%). The resistance rates of other selected pathogenic Gram-positive and Gram-negative bacteria are shown in Table 5 (Tab. 5) and Table 6 (Tab. 6).

### Antibiotic consumption

**Figure 5:**
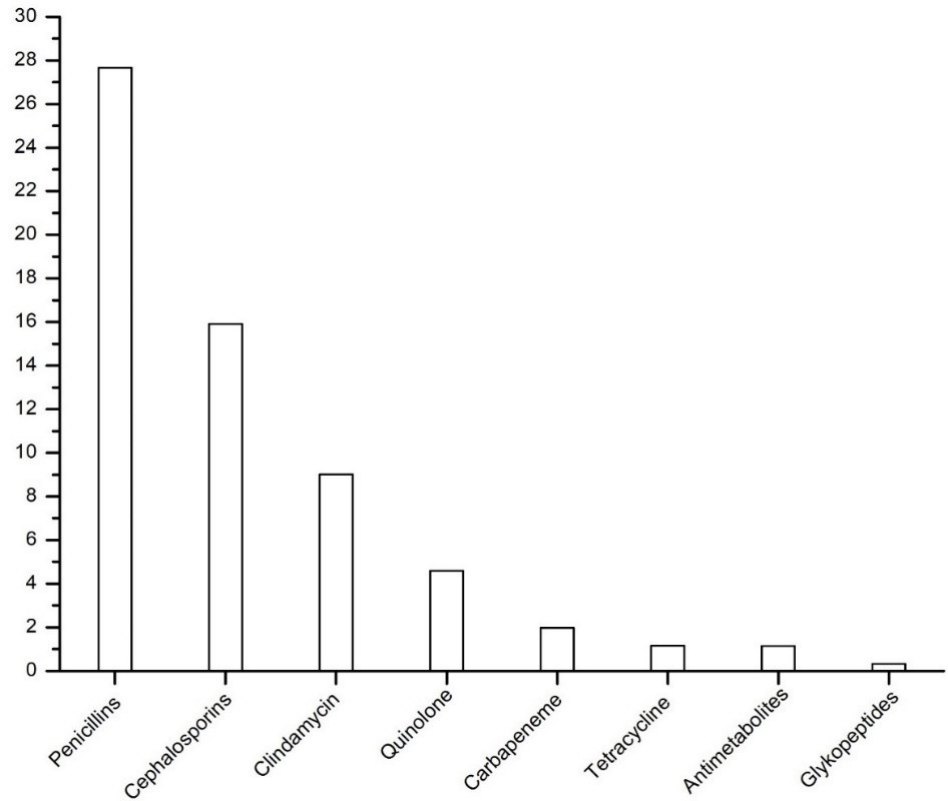
Total antibiotic consumption from 2011 to 2016

Total antibiotic consumption was highest in the penicillin group, followed by cephalosporins and clindamycin (Figure 5 (Fig. 5)). In detail, the aminopenicillins in combination with a BLI with 14.4 DDD/100 bed-days, followed by cefalexin with 10.8 and penicillin-G with 9.6 were the most frequently used antibiotics. The consumption of all antibiotic groups used in the course as well as the grouped cumulative antibiotic classes are shown in Table 7 (Tab. 7). Overall, in line with the relatively constant number of patients in connection with a relatively constant spectrum of germs and resistance, there were only minor variations in the consumption of antibiotics over time.

## Discussion

SSTIs are one of the most common indications for inpatient treatment in Dermatology [1], [2], [3], [4]. In our study sample, 12.4% of the principal diagnoses indicate a bacterial infection. When germs are usually not detectable antibiotic therapy is often initially empirical, which inevitably sometimes leads to treatment with unsuitable first-line therapy [19], [20]. Analysis of the *Retrospective Study to Assess the Clinical Management of Patients With Moderate-to-Severe Complicated SSTI or Community-Acquired Pneumonia in the Hospital Setting *(REACH) showed that in the absence of an early response (<72 h) to therapy in complicated SSTI often an infection with Gram-negative and anaerobic bacteria was present, whereas there was more of a Gram-positive spectrum with a rapid response to therapy [21]. Erysipelas is one of the most common bacterial infections in dermatology. Isolation of the mostly causal beta-hemolytic streptococci is not routinely carried out [22]. This explains the relatively low number of beta-hemolytic streptococci in our study.

If one compares the antibiotic consumption recorded in our study with values of the *Austria-wide resistance rate of the annual report on antibiotic resistance and consumption of antimicrobial substances in Austria* (AURES) for the same period, then here as there, beta-lactam antibiotics were the most commonly used group of substances, followed by quinolones (29.12–33.39 and 5.48–6.35 DDD/100 bed-days) [23]. The high consumption of the antibiotic groups described here, is due, among other factors, to adherence to the therapy recommendations for simple and complicated SSTIs of the *Infectious Diseases Society of America* [8]. 

Regarding resistance, data on critical pathogens are collected and evaluated annually through continuous monitoring at national, European and international level.

These microbiological data can be used to set targeted measures for antibiotic resistance. If one compares the annual *MRSA* rates of the AURES from 2011 to 2016 with ours, the individual values were at a similarly low level (2.2–8.2 vs. 7.1–9.1%) [23], [24]. There was no evidence of vancomycin or linezolid resistance within our study. When the *MRSA* rates of our study are combined with those of the *European Antimicrobial Resistance Surveillance* (EARS) network, the values of the study were well below the European median for the period 2011–2016 (13.7–18.6%). At this point it should be mentioned that in Europe there is a clear regional difference between the *MRSA* rates and their development between northern (e.g. 1.2% in 2016), southern and eastern Europe (e.g. 50.5% in 2016) with generally falling *MRSA* rates [25]. It is noteworthy that resistance to clindamycin has also been demonstrated with a high frequency in *methicillin-sensitive* *S. aureus*, which is particularly important when using this antibiotic empirically [26].

The resistance rates of *P. aeruginosa* in our study to aminoglycosides were higher than the Austria-wide rates (6.9–17.6 vs. 6.1–11.2%). The same was true for fluoroquinolones (5–45.6 vs. 7.2–18.5%) with a downward trend. The rate of resistance to ceftazidime was similar to the nationwide rate (2.8–21.7 vs. 9.5–14.1%). The resistance rate to piperacillin/tazobactam in our study showed a decreasing course except for 2012 and was lower than the Austrian average (1.6–43.5 vs. 13.2–17.5%). In the group of carbapenems, the resistance rate in our study was similarly low as in the Austria-wide comparison (2.5–21.6 vs. 13.3–16.2%) [23], [24]. Global data from the *Study for Monitoring Antimicrobial Resistance Trends* (SMART) from 2002–2011 showed resistance rates of 20–40% for imipenem in bacterial isolates from intra-abdominal and urogenital infections [27]. In addition, the resistance rates for fluoroquinolones in a study carried out in North America increased from 22% to 33% from 2005–2010 and the rates for imipenem, piperacillin/tazobactam, cefepime and ceftazidime remained stable at 20–26% [28]. Compared to international results, our figures show that at our institution these germs are currently easier to treat.

Looking at the resistance rates of *E. coli* regarding aminopenicillins and third generation cephalosporins, they were below the Austria-wide rate (4.1–55.9 vs. 49.9–51.3%; 0 vs. 9.0–10%; respectively). No difference was detected for the fluoroquinolones (16.9–22.6 vs. 19.8–22.2%). In the Austrian comparison, the resistance rates for aminoglycosides were similarly low (4.1–13.6 vs. 6.3–7.8%) [23], [24]. Data from the *Meropenem Yearly Susceptibility Test Information Collection *(MYSTIC) study from 1997–2000 in relation to Europe generally showed a higher frequency of *ESBL-producing E. coli* in southern and eastern European countries and the same resistance rates for carbapenems (0 vs. 1.1%) and aminoglycosides (33 vs. 31%) [29].

The limitations of our study are mainly due to the retrospective design. On the one hand, it was only possible to a limited extent to determine the influence of potential changes in the disease spectrum of the patient collective on the germ spectrum. However, the relative homogeneity of the available data makes such an influence unlikely. Furthermore, it cannot be ruled out that observed changes (e.g., in antibiotic consumption and in the frequency and way smears were taken) are caused by different medical assessments and decisions by individual doctors and nursing staff. Other factors that may have influenced the results, but could not be recorded by us, are previous antibiotic therapies and the failure to differentiate between community-acquired and nosocomial germs. Furthermore, contaminants from the skin flora (coagulase-negative staphylococci, corynebacteria and possibly also enterococci) could not be reliably excluded.

Seen globally, the increasing resistance to antibiotics has far-reaching consequences through the limitation of treatment options for infections and through increased morbidity, mortality and costs [30]. There is sufficient evidence for nosocomial infections showing that continuous monitoring of infection rates and resistance behavior leads to an improvement in the quality of patient care [31]. Such monitoring has not been done in dermatology before. The results of this retrospective study offer the opportunity to get an up-to-date overview of the bacterial epidemiology of a dermatological inpatient ward and to observe changes in the bacterial spectrum and antibiotic consumption. 

## Conclusions

The results of the study confirm

1. the continuous relevance of *S. aureus* and *P. aeruginosa* in skin disease,
2. the low prevalence of multi-resistant germs, and
3. a variation in the mostly empirical consumption of antibiotics depending on availability and prescription behavior at our institution.

Regular microbiological analysis can be an important instrument for antibiotic stewardship also in dermatological departments.

## Notes

### Competing interests

The authors declare that they have no competing interests.

## Figures and Tables

**Table 1:**
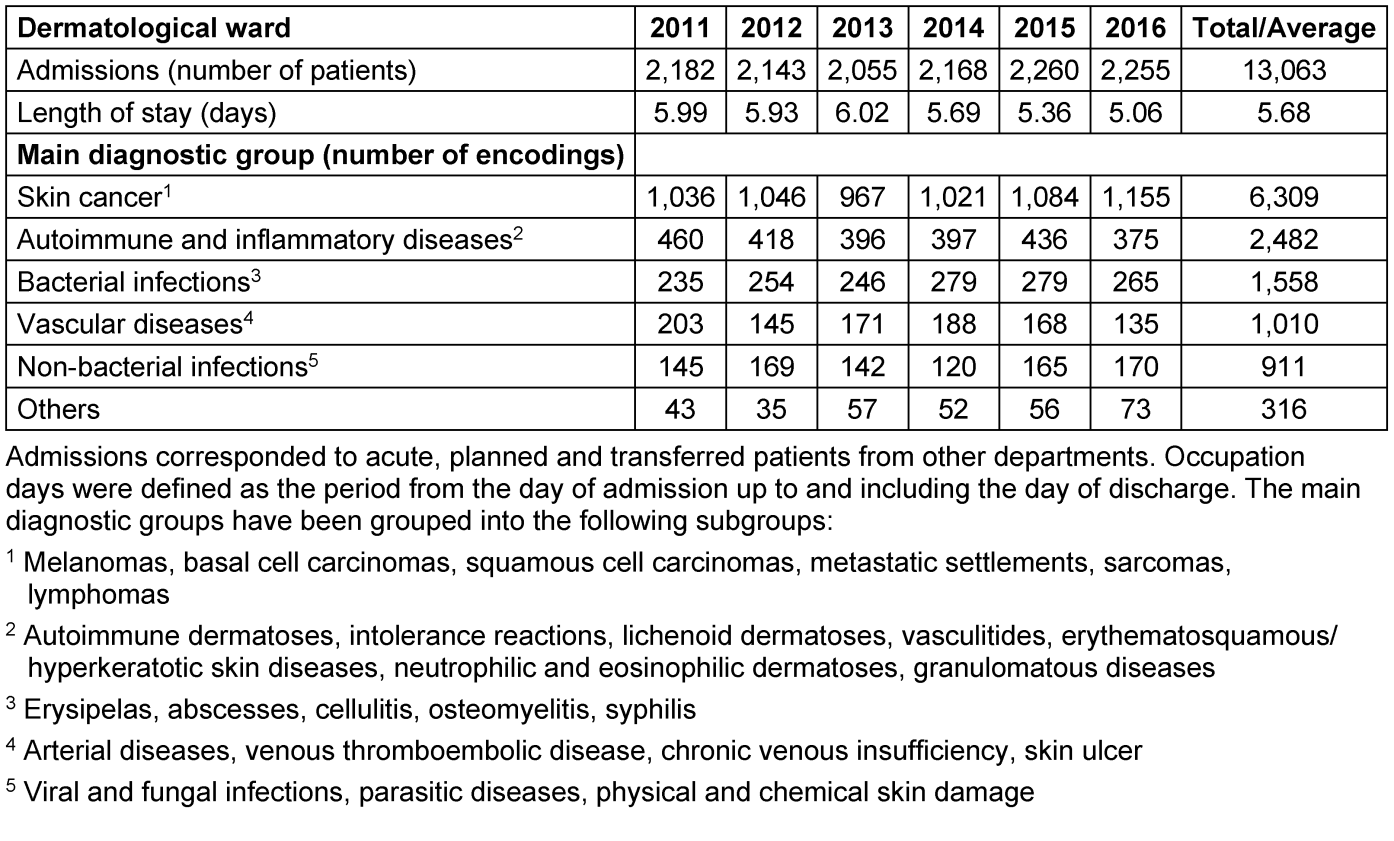
Demographic characteristics of dermatological inpatients

**Table 2:**
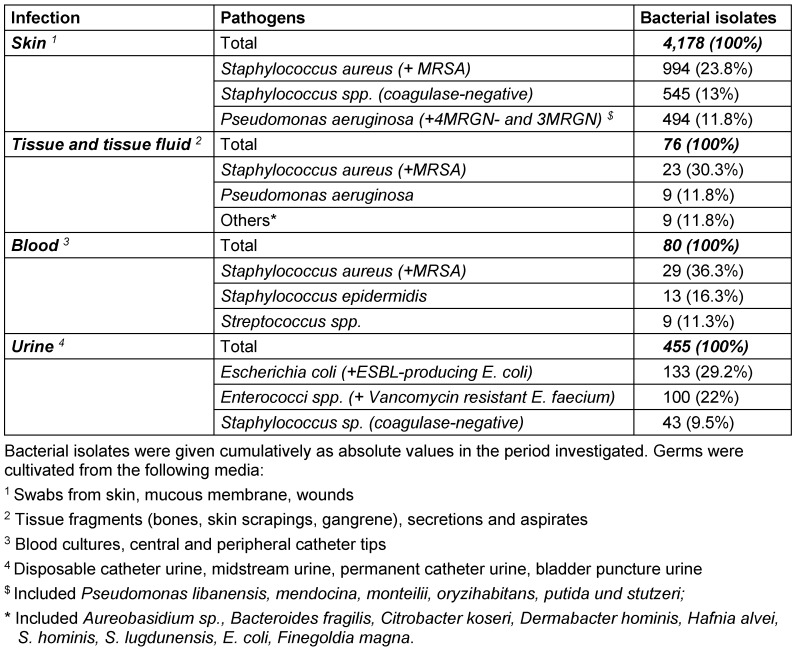
The three most common bacterial strains separated by origin

**Table 3:**
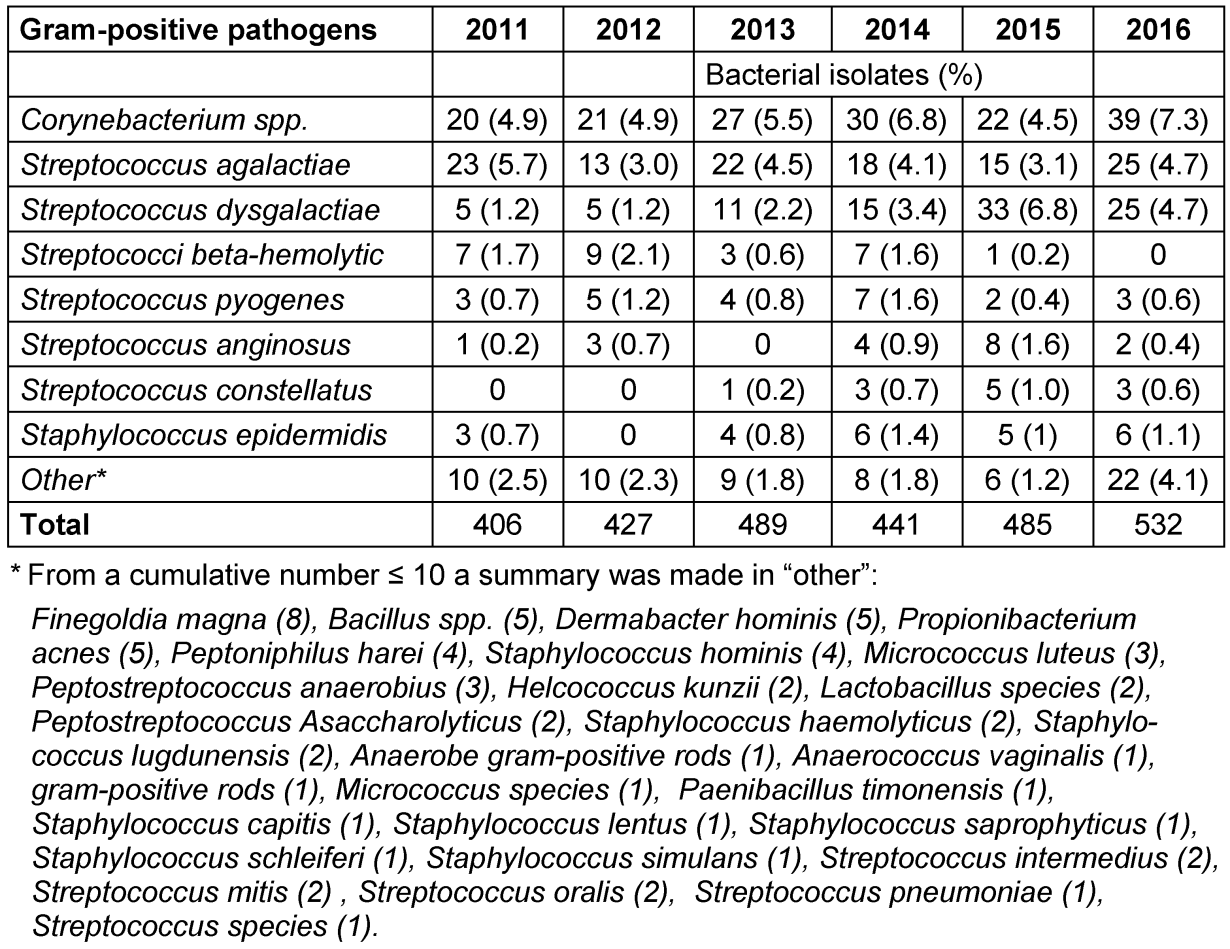
Other common gram-positive bacterial strains from 2011–2016

**Table 4:**
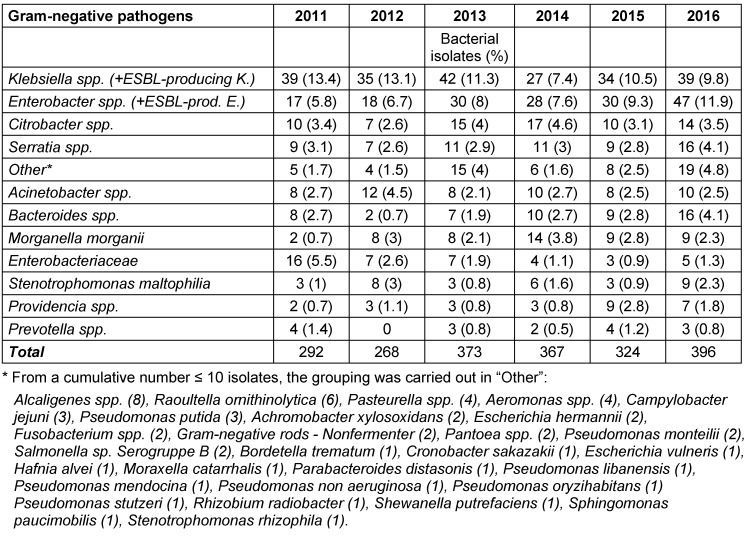
Other common gram-negative bacterial strains from 2011–2016

**Table 5:**
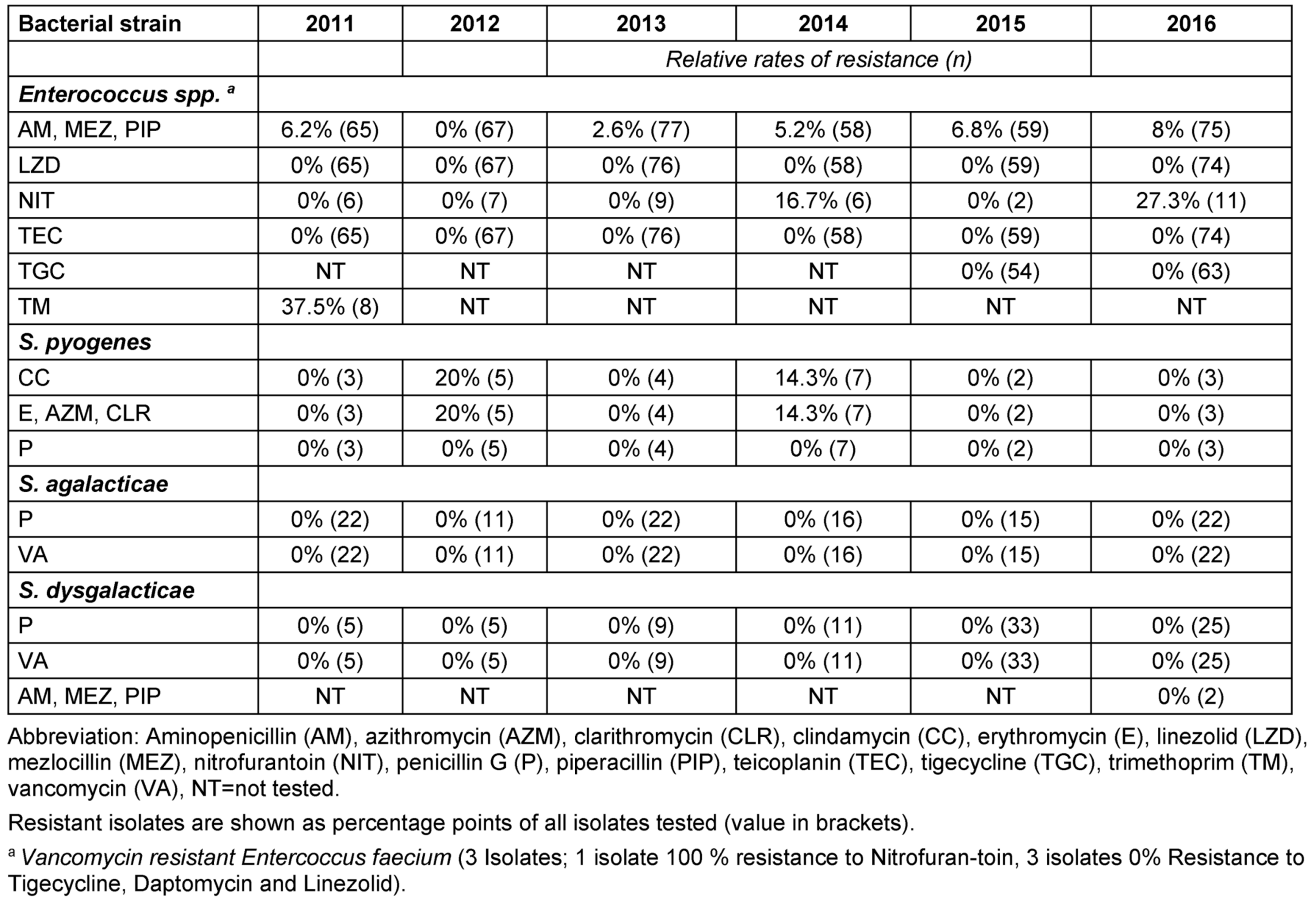
Resistance rates of other important gram-negative pathogenic germs from 2011 to 2016

**Table 6:**
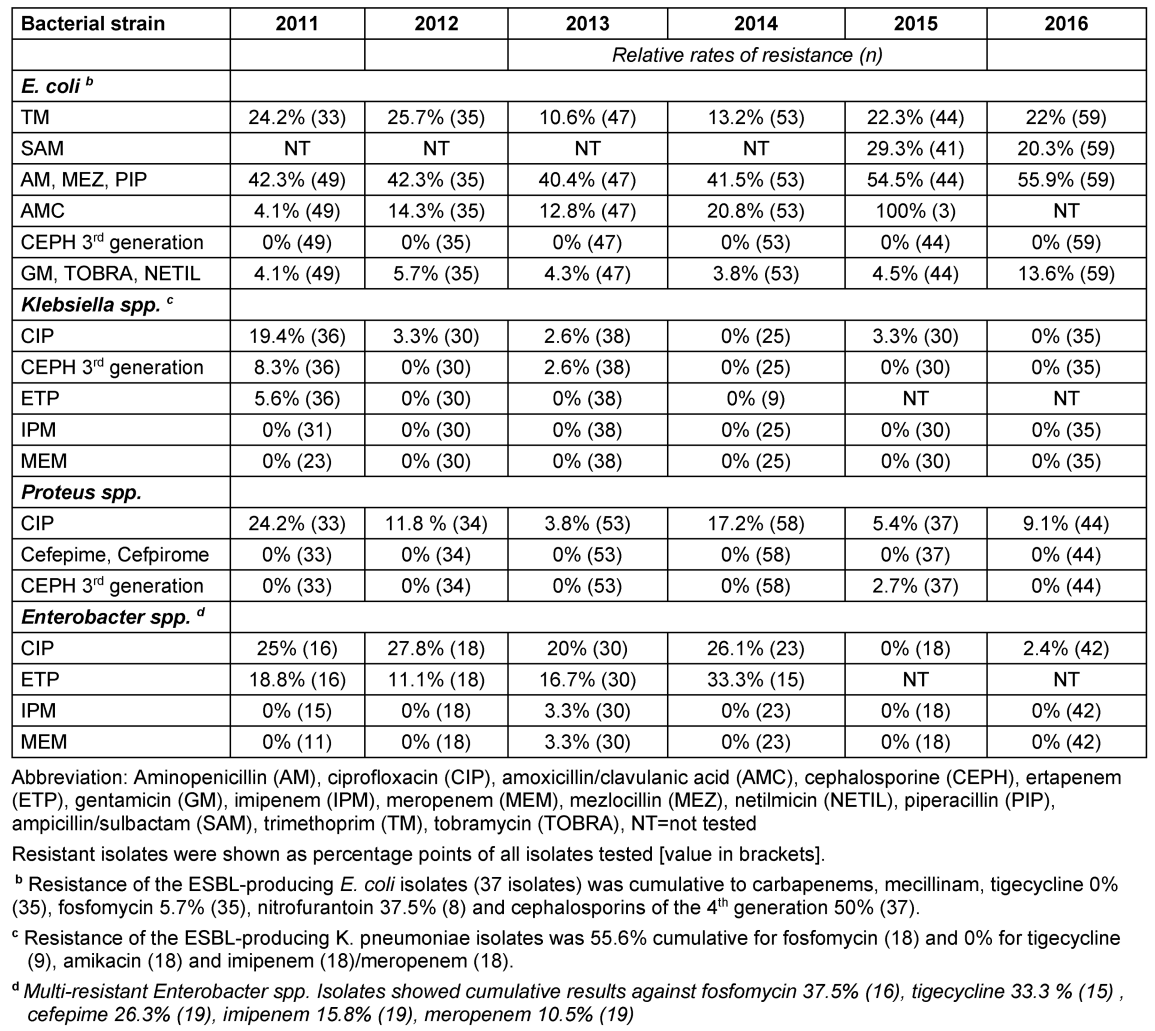
Resistance rates of other important gram-negative pathogenic germs from 2011 to 2016

**Table 7:**
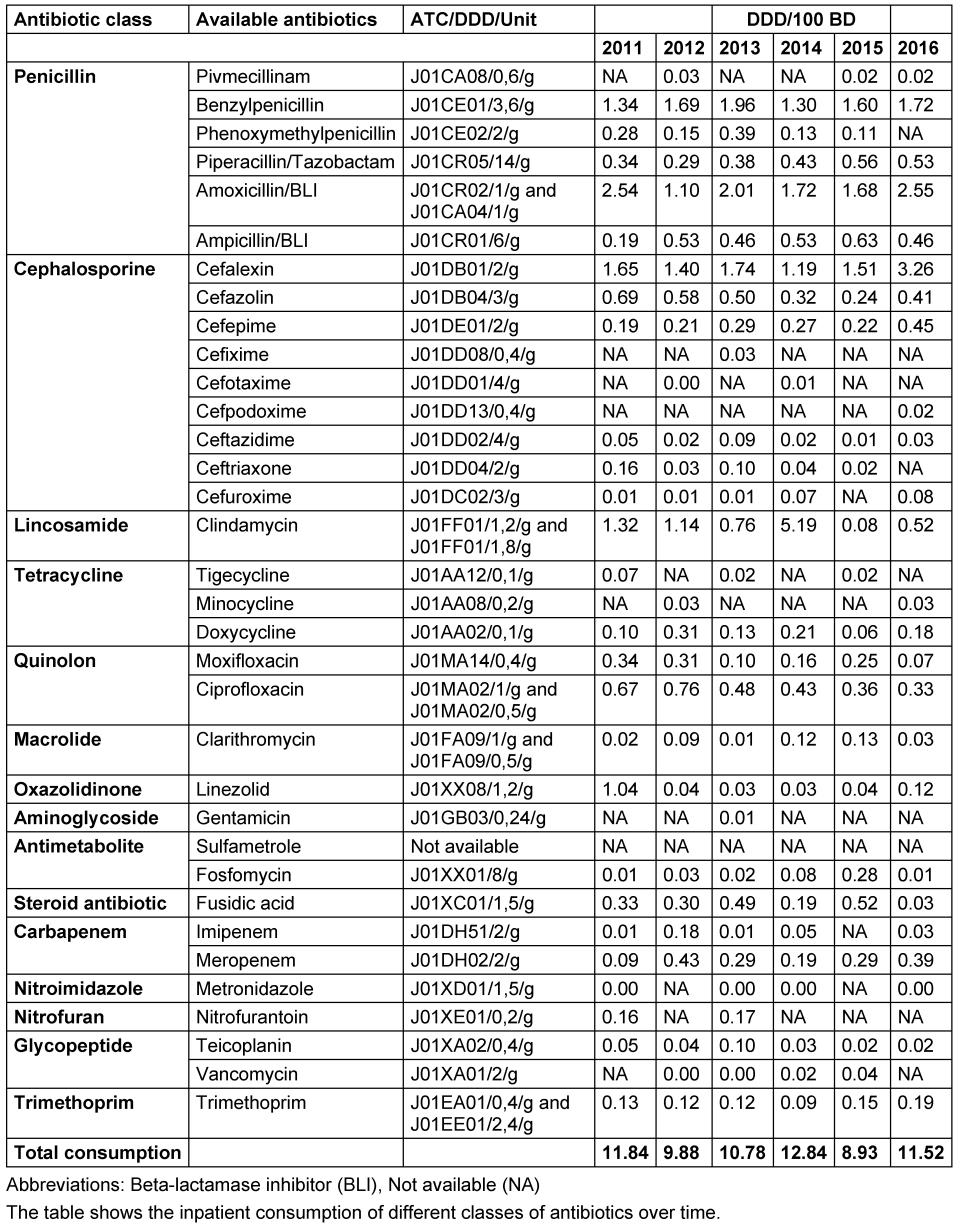
Consumption behavior for ATC code J01 antibiotics in the course of 2011 to 2016
